# Effet of Combined Nitrogen Dioxide and Carbon Nanoparticle Exposure on Lung Function During Ovalbumin Sensitization in Brown Norway Rat

**DOI:** 10.1371/journal.pone.0045687

**Published:** 2012-09-28

**Authors:** Skander Layachi, Françoise Rogerieux, Franck Robidel, Ghislaine Lacroix, Sam Bayat

**Affiliations:** 1 University of Picardie Jules Verne, EA4285 Laboratoire Périnatalité et Risques Toxiques- UMI01 INERIS, Amiens, France; 2 Departments of Paediatric Cardiology and Respiratory Medicine, Paediatric Lung Function Laboratory, Amiens University Hospital, Amiens, France; 3 Institut National de l’Environnement Industriel et des Risques (INERIS), Verneuil-en Halatte, France; Cincinnati Children’s Hospital Medical Center, United States of America

## Abstract

The interaction of particulate and gaseous pollutants in their effects on the severity of allergic inflammation and airway responsiveness are not well understood. We assessed the effect of exposure to NO_2_ in the presence or absence of repetitive treatment with carbon nanoparticle (CNP) during allergen sensitization and challenges in Borwn-Norway (BN) rat, in order to assess their interactions on lung function and airway responses (AR) to allergen and methacholine (MCH), end-expiratory lung volume (EELV), bronchoalveolar lavage fluid (BALF) cellular content, serum and BALF cytokine levels and histological changes. Animals were divided into the following groups (n = 6): Control; CNP (Degussa-FW2): 13 nm, 0.5 mg/kg instilled intratracheally ×3 at 7-day intervals; OVA: ovalbumin-sensitised; OVA+CNP: both sensitized and exposed to CNP. Rats were divided into equal groups exposed either to air or to NO_2_, 10 ppm, 6 h/d, 5d/wk for 4 weeks. Exposure to NO_2_, significantly enhanced lung inflammation and airway reactivity, with a significantly larger effect in animals sensitized to allergen, which was related to a higher expression of TH1 and TH2-type cytokines. Conversely, exposure to NO_2_ in animals undergoing repeated tracheal instillation of CNP alone, increased BALF neutrophilia and enhanced the expression of TH1 cytokines: TNF-α and IFN-γ, but did not show an additive effect on airway reactivity in comparison to NO_2_ alone. The exposure to NO_2_ combined with CNP treatment and allergen sensitization however, unexpectedly resulted in a significant decrease in both airway reactivity to allergen and to methacholine, and a reduction in TH2-type cytokines compared to allergen sensitization alone. EELV was significantly reduced with sensitization, CNP treatment or both. These data suggest an immunomodulatory effect of repeated tracheal instillation of CNP on the proinflammatory effects of NO_2_ exposure in sensitized BN rat. Furthermore, our findings suggest that NO_2_, CNP and OVA sensitization may significantly slow overall lung growth in parenchymally mature animals.

## Introduction

Asthma is a major public health problem, affecting 300 million people worldwide, and has increased considerably in prevalence over the past three decades, particularly in many developed countries [1]. Although the causes for this increase are not well known, a considerable amount of epidemiological evidence suggests that certain components of air pollution such as ozone, nitrogen dioxide (NO_2_), and particulate matter (PM), as well as a variety of allergens may play important roles [2], [3]. Particulate matter and NO_2_ are important ambient air pollutants, their health effects have been extensively reviewed, air quality standards and guidelines have been proposed to protect public health.

NO_2_ is present in the outdoor environment, resulting from emissions from automobile exhausts, where its level can reach 4 ppm during heavy traffic [4], [5]. In the indoor environment, the concentration of NO_2_ produced by sources such as kerosene heaters and gas cookers often exceeds outdoor concentrations and reach peak values above 4 ppm [4], [6]. This pollutant has been associated with an increased morbidity rate for respiratory disease [7]. In fact, exposure to low levels of NO_2_ may alter respiratory function in several animal species and increase airway responsiveness to contractile agents. Also structural damages are induced by acute and chronic exposure resulting in lung inflammation and pulmonary fibrosis. The biochemical alterations which occur in the respiratory system following exposure to the oxidant gas include changes in lung lipids, antioxidant metabolism and enzyme activity [8].

The exposure to PM can lead to lung function impairment and exacerbation of pre-existing diseases, particularly allergic asthma [9]. Exposure to PM promotes lung inflammation through oxidative stress and lipid peroxidation [10]. Diesel exhaust particles (DEP) are the main component of PM. Previous studies have demonstrated that DEP can worsen both lipopolysaccharide-induced lung inflammation, representing an innate immunity [11], and allergic lung inflammation [12], [13]. Evidence in the literature suggests that the effect of particles on allergic inflammation may be modified by the properties of the particle surface, such as adsorbed organic chemicals and metals and possibly other factors such as surface charge, structure and size [14]. In this respect, carbon black nanoparticles (CNP) have previously been used as a surrogate of the physical core of nanoparticular combustion-derived PM. Smaller (13 nm) sized CNP have been shown to produce a stronger aggravation of allergic inflammation than larger (56 nm) particles in mouse [15].

T helper 2 (TH2) cells have a central role in the inflammatory response in allergic asthma, by releasing interleukin-4 (IL-4) and IL-13, both of which stimulate B cells to synthesize IgE, and IL-5 which are necessary for eosinophilic inflammation [16], while suppressing the production of TH1 cell cytokines, e.g.: IFN-γ and IL-2 [17], [18]. On the other hand, bronchial and alveolar epithelial cells and macrophages are the initial cells to interact with ambient particles. Epithelial cells produce cytokines such as IL-1, IL-6, IL-8, and TNF-α, whereas macrophages release cytokines such as TNF-α and IL-8 [14], [19]. How exactly the interaction of particulate and gaseous pollutants may orient the immune system towards a TH1- or TH2-like response and ultimately affect the severity of allergic lung inflammation and airway reactivity is a matter of speculation. Few reports in the literature have focused on the effects of combined exposure to nanoparticles and gaseous air pollutants, and to our knowledge, there are no data on the effect of combined NO_2_ and CNP exposure in experimental animal models of allergic asthma.

The aim of this study was therefore to assess the effect of exposure to NO_2_ in the presence or absence of repetitive treatment with CNP during allergen sensitization and challenges in BN rat, in order to examine the effects of these exposures and their interactions on lung function and airway responses to both specific (i.e. allergen) and non-specific, (i.e. methacholine) airway reactivity, end-expiratory lung volume (EELV), bronchoalveolar lavage fluid (BALF) cellular content, serum and BALF cytokine levels and histological changes.

## Materials and Methods

### Study Protocol

Animal care and experimental procedures were in accordance with the Guidelines for the Care and Use of Animals published by the American Physiologic Society and approved by the local institutional ethics authorities (Comité d’Éthique Restreint de l’Institut National de l’Environnement Industriel Et des Risques). All efforts were made to minimize suffering during the various procedures. Male Brown Norway (BN) rats, aged 7 weeks, weighing 140–160 g (Charles River Laboratories, l’Arbresle, France) were housed in a clean air room with restricted access. The animals had free access to food and water, and were allowed to acclimate for 1 week before the experiments.

**Figure 1:**
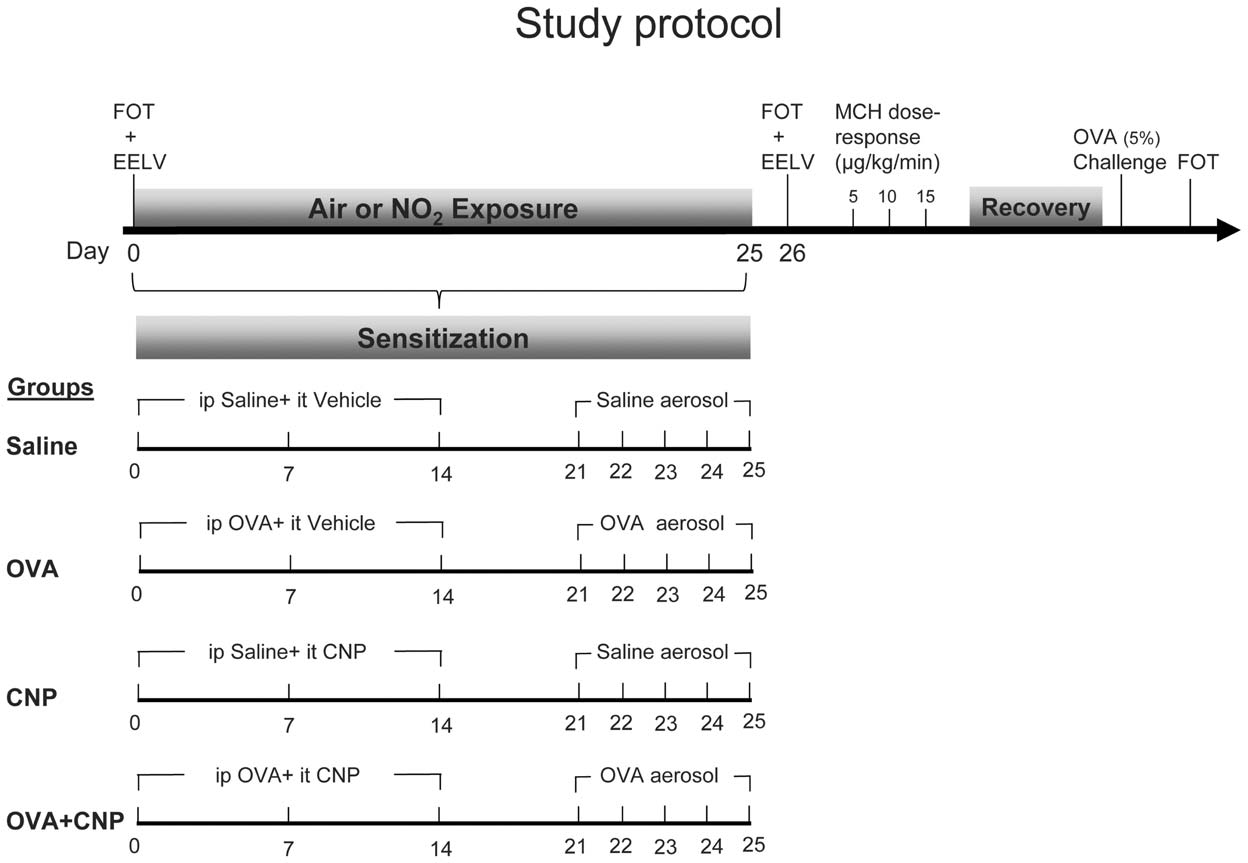
Study protocol. OVA: ovalbumin; CNP: Degussa-FW2 (13 nm) carbon nanoparticles; MCH: methacholine; FOT: forced oscillation respiratory mechanics measurements; EELV: end-expiratory lung volume measurements. Brown-Norway rats underwent 4 treatments; OVA-sensitization, CNP tracheal instillation, Both OVA-sensitization and CNP treatment, and sham sensitization and tracheal instillation of vehicle. Equal treatment groups were exposed to either air or NO_2_.

The study protocol is illustrated in Figure 1. The animals were divided into 4 groups (n = 16 per group): Control, carbon nanoparticle treatment (CNP), ovalbumin-sensitization (OVA), and combined sensitization and exposure to nanoparticles (OVA+CNP). Rats were divided into equal groups (n = 8) exposed to either air or to NO_2_, 10 ppm, 6 h/d, 5d/wk for 4 weeks. 24 hours after the end of the exposure period, animals were anesthetized and mechanically ventilated, EELV was measured using whole-body plethysmography. Airway and lung tissue mechanical parameters were measured using forced oscillation technique (FOT) at baseline and after intravenous infusion of methacholine (MCH) at 5, 10 and 15 µg/kg/min (γ), after recovery, and following allergen inhalation. Terminal bronchoalveolar lavage fluid (BALF) and serum samples were obtained. Two rats per group were used for lung histological analysis.

### Allergen Sensitization and Challenge

The sensitization and challenge protocols were similar to previously published models [20], [21], with some modifications as described below. On days 0, 7, and 14; rats were actively sensitized by intraperitoneal injection of 1 mg Ovalbumin (OVA) (Grade V, Sigma-aldrich, St Quentin, France) and 100 mg aluminum hydroxide, (Sigma-aldrich, St Quentin, France) in 1 ml of normal saline. Rats were challenged using aerosolized 1% OVA in endotoxin-free saline for 30 min on days 21 to 25 using an ultrasonic nebulizer (Systam LS 290, Villeneuve sur Lot, France) with an output of 0.2 ml/min. Control rats were sham-sensitized and challenged with identical amounts of saline solution.

### NO_2_ Exposure

We used a custom-made stainless steel and glass exposure exposure system, developed in the pulmonary toxicology laboratory of the Institut National de l’Environnement Industriel et des Risques. Unrestrained and conscious rats were placed in four 20 L cylindrical exposure chambers, and exposed to 10±0.1 ppm NO_2_ (Air Liquide, Loos, France) or filtered air, with a flow rate of 5l/min, during the sensitization and CNP treatment period, 6 h/d, 5d/week, for 4 weeks. The flow of NO_2_ or air in each chamber was monitored by a mass flow-meter during the period of exposure. The pressure of the glass exposure chamber was controlled by a manometer.

### Carbon Nanoparticle Treatment

On days 0, 7, and 14; rats were lightly anesthetized using intramuscular ketamine hydrochloride (0.5 mg/kg, Imalgène, Lyon, France), atropine (0.1 mg/kg, Lavoisier, Paris, France) and xylazine (1 mg/kg, Bayer Animal Health, Leverkusen, Germany) and instilled intratracheally with 0.5 mg/kg of carbon nanoparticle (Degussa-FW2∶13 nm, Evonik Industries, Essen, Germany) or vehicle in 150 µl of vehicle. The dosage of CNP was based on Li et al. [22]. CNP were suspended in saline. The suspensions were sonicated 10 min at 40 W with an ultrasonic probe (sonicator ultrasonic processor XL 2020, Misonix incorporated). Using this dispersion method, more than 50% of total agglomerates have a size smaller than 10 µm that corresponds to breathable agglomerates, as measured by laser scattering (Mastersizer X, Malvern instruments, Malvern, UK).

### Airway and Tissue Mechanics

Twenty four hours after the end of the exposure period, the rats were anesthetized by intraperitoneal injection of chloral hydrate (400 mg/kg). The trachea was intubated with a polyethylene cannula (14-gauge, Braun, Boulogne Billancourt, France) and the rats were mechanically ventilated with a tidal volume (VT) of 10 ml/kg body weight, a respiratory rate of 70–80/min. The end-expiratory pressure was set to zero. Anesthesia was maintained with hourly supplemental doses of intraperitoneal chloral hydrate (150 mg/kg). The carotid artery was cannulated with a 22G catheter (Abbocath, Hospira, Meudon-La-Foret, France) and attached to a pressure transducer (ADInstruments, Oxford, United Kingdom) for continuous blood pressure monitoring. The right jugular vein was cannulated with a 26G catheter (Apotechnia, Aubagne, France) connected to an infusion pump (Injectoma Agila, Fresenius Kabi, Sèvres, France) for continuous infusion incremental doses of 5, 10 and 15 µg/kg/mn (γ) of methacholine.

To characterize the airway and tissue mechanics, the input impedance of the respiratory system (*Zrs*) was measured using the forced oscillatory technique, as described in detail previously [23], [24]. Briefly, the tracheal cannula was connected to a loudspeaker-in-box system at end-expiration. The loudspeaker generated a small-amplitude pseudorandom signal with frequency components between 0.5 and 21 Hz through a polyethylene wave-tube (L = 100 cm, ID = 2 mm). Two identical pressure transducers (model 33NA002D, ICSensors, Milpitas, CA) were used for measurement of the lateral pressures at the loudspeaker and at the tracheal end of the wave-tube. *Zrs* was calculated as the load impedance of the wave-tube [25]. In order to separate the airway and tissue parameters, a model was fitted to the *Zrs* spectra by minimizing the relative differences between the measured and modeled impedance values. The model contained a frequency-independent airway resistance (Raw) and inertance (Iaw) in series with a constant-phase tissue compartment characterized by the coefficients of tissue damping (G) and elastance (H) [26]. The impedance of the tracheal cannula and the connecting tubing was also determined, and Raw and Iaw were corrected by subtracting the instrumental resistance and inertance values from them. The respiratory, hemodynamic parameters and rectal temperature were continuously monitored and recorded using a data collection and acquisition system (PowerLab ADinstrument, Oxford, UK).

### Measurement of EELV

EELV measurements were performed with a body plethysmograph, as detailed previously [27]. Briefly, the trachea was occluded at end expiration until 3–4 spontaneous inspiratory efforts were generated by the animal in the closed box. Changes in tracheal pressure (Ptr) and plethysmograph box pressure (Pb) were recorded during these maneuvers, and EELV was calculated by applying Boyle’s law to the relationship between Ptr and Pb after correction for the box impedance [27]. The EELV was assessed before any exposure or treatment in 7 week-old naïve rats (n = 32) and following NO_2_ and sham exposures at age 11 weeks, in all animals.

### BAL

Bronchoalveolar lavage (BAL) was performed after the measurements of EELV, respiratory mechanics and airway responsiveness. Briefly, through the tracheal cannula, the lungs were washed 3 times with 9 ml of sterile Phosphate buffered saline. The BAL fluid (BALF) was centrifuged (5 min, 150 g at 4°C), and the cell-free BAL was concentrated by further centrifugation (2000 g, 4°C) in Amicon Ultra tubes (Millipore, Molsheim, France) to a final volume of 1 ml. Protein concentration was assessed using the Bradford method [28]. The BAL cell pellets were resuspended in 1 ml RPMI 1640 medium (Gibco, Villebon sur Yvette, France). The total cell numbers were counted automatically (Coulter Counter ZM, Coultrinics, Margency, France). Cells were then applied to slides by Cytospin centrifugation (Shandon Cytospin 2, Pittsburgh, PA) at 300 rpm for 5 min. Afterwards, May-Grünwald Giemsa staining was performed, and a total of 300–500 cells were counted for each sample by light microscopy.

### Cytokine and IgE Analysis

TH1 and TH2 cytokine analysis was performed using a multi-array immunoassay, on the concentrated BALF and serum samples (MULTI-SPOT Rat Demonstration 7-Plex Assay Ultra-Sensitive Kit; Meso Scale Discovery, Gaithersburg, MD, USA) as described previously [29]. The following cytokines were measured: IL-1β, KC/GRO (keratinocyte chemotractant/growth-related oncogene), TNF-α, IFN-γ, IL-4, IL-5 and IL-13. The concentrations of cytokines were quantified using an 8-point calibration curve constructed from a plot of the signal intensity for a series of known concentrations of the multiplex standard provided by the kit manufacturer. The data were analyzed using MSD Workbench software. An MSD Sector Imager (Sector Imager 6000; Gaithersburg, MD, USA) was utilized to read the plates. Cytokines in BALF and plasma sample were quantified in duplicate according to the manufacturer’s protocol. The IL-17A levels were quantified in BALF by ELISA (Rat IL-17A Platinum Elisa, eBioscience, Bender Medsystems GMBH, Vienna, Austria). Serum total IgE levels were measured using an ELISA immunoperoxidase assay (GenWay Biotech, San Diego, CA).

### Lung Histology

In order to study lung inflammation and airway remodeling, lung histopathology was assessed in 2 rats per group, 24 h after the challenges. The lungs were fixed in 10% formalin at 25 cmH_2_O and paraffin-embedded. Histological slides were prepared from 5 µm mid transversal para-hilar and lower sections to sample large, medium and small airways. The tissue sections were stained with hematoxylin, eosin and saffron (HES) for general morphology and dispersion of carbon nanoparticles. To assess goblet cell hyperplasia, sections were stained with Periodic-Acid-Schiff (PAS, Sigma Chemicals, St Louis, MO). Lung sections were also examined with Masson’s trichrome stain (Sigma-Aldrich, Saint-Quentin Fallavier, France) to assess deposition of peribronchial and perivascular collagen.

### Statistical Analysis

Data are presented as means ± SE. The statistical analysis was performed using SigmaPlot version 10 Software, (SigmaPlot, Systat Software, San Jose, CA, USA). Airway and tissue mechanics parameters, MCH dose-responses for Raw, G, H, the changes in EELV, the changes in BALF cytology and BAL fluid and serum cytokines were assessed by repeated-measures ANOVA followed by a Student-Newman-Keuls multiple comparisons procedure in the four treatments groups (Saline, CNP, OVA, and OVA+CNP) and two conditions (Air or NO_2_ exposure). A p-value of <0.05 was considered as significant.

## Results

### Changes in Airway and Tissue Mechanics and Response to MCH and Allergen Provocation

**Figure 2:**
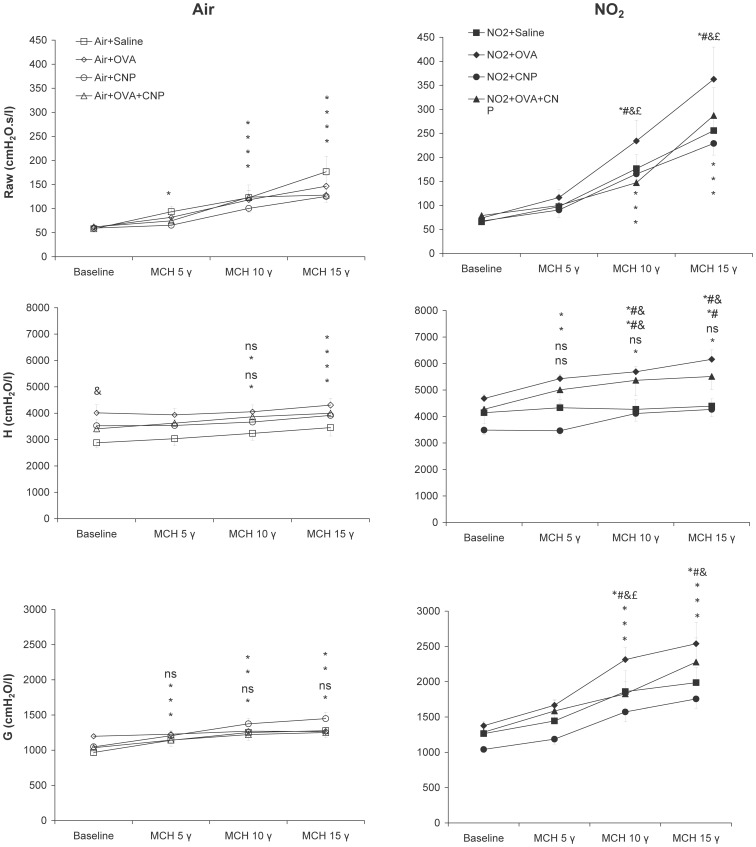
Changes in airway (Raw) and tissue (G and H) mechanical parameters in response to increasing doses of intravenously infused methacholine (MCH). Data are means ± SE (n = 6 per group); *: p<0.05 vs. Baseline within condition; #: p<0.05 vs. Air+OVA; &: p<0.05 vs. Saline within condition; £: p<0.05 vs. NO2+OVA+CNP, by repeated-measures ANOVA. Responsiveness to MCH significantly increased following NO_2_ exposure, with significant differences among the subgroups: responsiveness was substantially larger in the NO2+OVA group. Animals treated with CNP during sensitization and exposure to NO_2_ (NO2+OVA+CNP) had significantly lower MCH responses than the non CNP-treated counterparts.

Figure 2 shows the respiratory mechanical parameters at baseline and during MCH challenge in air and NO_2_-exposed animals. In the air-exposed animals, tissue elastance; H was significantly increased in the OVA sensitized group at baseline (p = 0.013), while Raw and G did not significantly change. No significant differences in the respiratory mechanical parameters were observed in the CNP-treated vs. control animals. Four weeks of exposure to NO_2_ did not produce significant changes in Raw, G and H, at baseline; i.e.: prior to MCH provocation.

Infusion of MCH significantly increased both airway (Raw) and tissue (H and G) mechanical parameters in the air-exposed animals, without significant differences in responsiveness in between groups. Responsiveness to MCH however, was significantly increased following NO_2_ exposure, with significant differences among the subgroups. Airway responsiveness was substantially larger in the NO_2_+OVA group. The increases in Raw, G and H with both 10 and 15 µg/kg/min of MCH, were significantly higher than the Air+OVA group and than the other NO_2_-exposed groups. Animals treated with CNP during sensitization and exposure to NO_2_ (NO_2_+OVA+CNP) had significantly lower MCH responses than the non-treated counterparts (Figure 2A).

**Figure 3:**
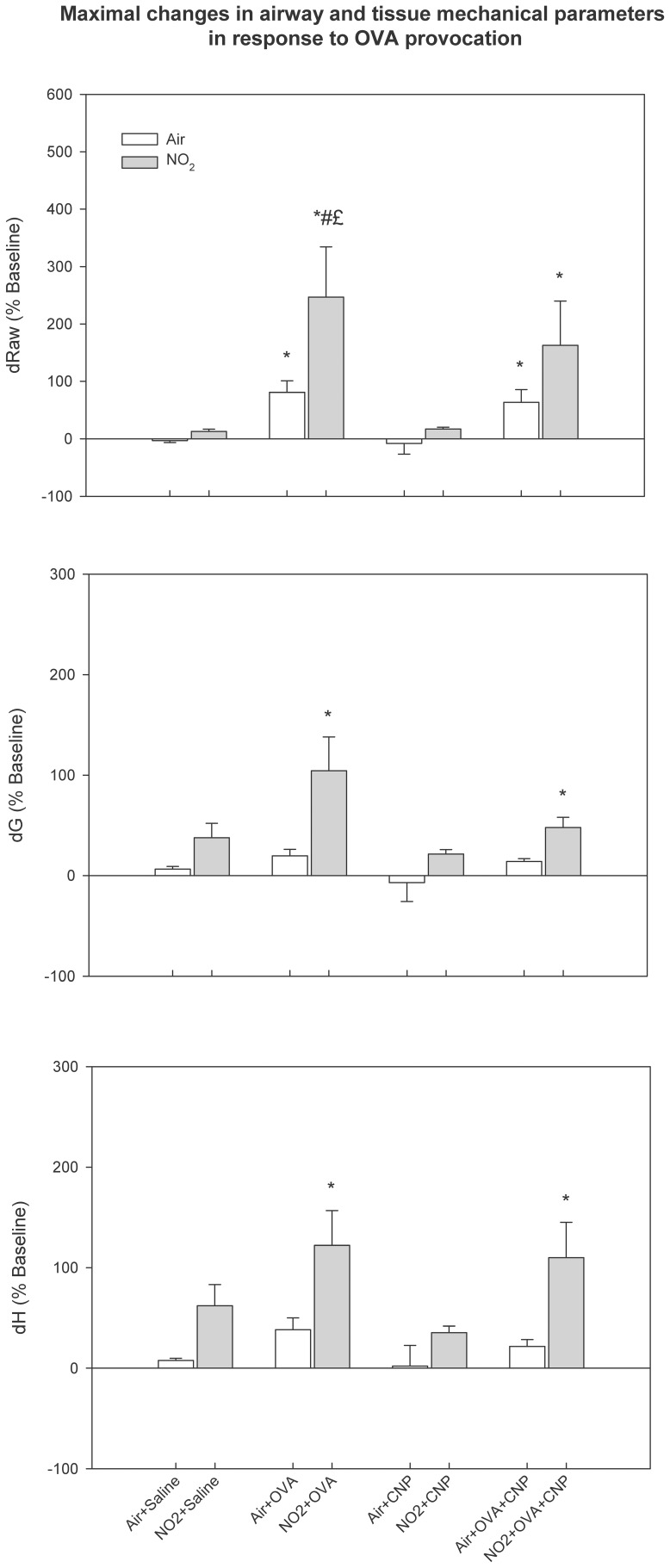
Maximal airway and tissue responses to OVA provocation, relative to the recovery level from MCH. Data are means ± SE (n = 6 per group); *: p<0.05 vs. Air+Saline within condition; #: p<0.05 vs. Air+OVA; £: vs. Air+OVA+CNP, by ANOVA. The responses in Raw were significantly larger in the NO_2_-exposed and sensitized animals. In the group concomitantly treated with CNP during sensitization (NO2+OVA+CNP), the maximal Raw change in response to OVA provocation was smaller compared to NO2+OVA.

Maximal airway and tissue responses to OVA provocation, relative to the recovery level from MCH, are summarized in Figure 3. Significant changes in Raw, G and H, were observed in the sensitized groups. The responses in Raw were significantly larger in the NO_2_-exposed animals. In the group concomitantly treated with CNP during sensitization (NO_2_+OVA+CNP), the maximal Raw change in response to OVA provocation was smaller compared to the NO_2_-OVA group (p<0.05). The parameters that reflect the lung peripheral mechanics; G and H increased significantly only in the sensitized NO_2_-exposed animals.

### EELV

**Figure 4:**
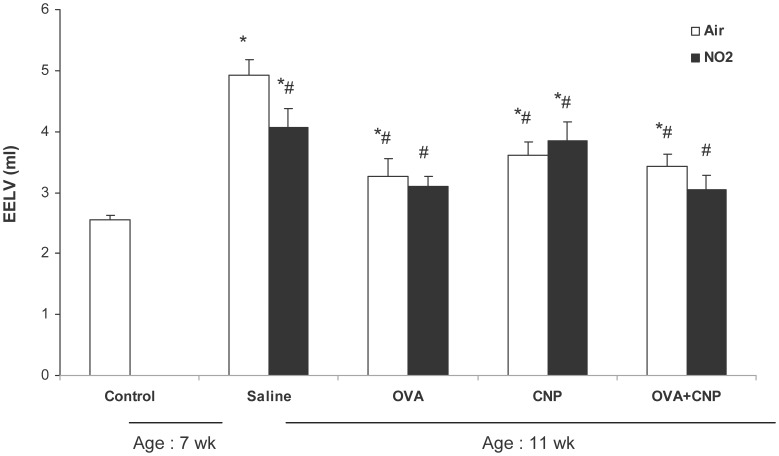
Changes in end-expiratory lung volume (EELV). Data are means ± SE (n = 6 per group); *: p<0.05 vs. 7 week old Control; #: p<0.05 vs. 11-week old Air+Saline by ANOVA. The EELV was significantly lower in the NO2+Saline vs. Air+Saline animals. Also, EELV was significantly reduced by sensitization to allergen, CNP treatment or both, independent of exposure to NO_2_. Comparison of EELV at 11 weeks vs. non-exposed controls at 7 weeks (n = 32) revealed that EELV significantly increased over the 4-week exposure period in all groups with the exception of NO2+OVA and NO2+OVA+CNP.

Figure 4 summarizes the EELV data. EELV was significantly reduced with sensitization, CNP treatment or both, independent of exposure to NO_2_. EELV was also significantly decreased in the NO_2_-Saline vs. Air-Saline animals. Comparison of EELV at 11 weeks vs. non-exposed controls at 7 weeks (n = 32) revealed that EELV significantly increased over the 4-week exposure period in all groups with the exception of NO_2_+OVA and NO_2_+OVA+CNP. After normalization to body weight, in order to account for variations in this parameter with time and in-between groups, a significant increase in EELV (p = 0.006) was observed only in the Air+Saline group.

### BAL Inflammatory Cells

The results of cell counts in BAL are summarized in Table 1. Exposure to NO_2_ did not change significantly the profile of BAL cytology. OVA-sensitizaion significantly increased eosinophil counts (p<0.05) without a significant difference following NO_2_ exposure**.** However, NO_2_-exposed animals that received CNP treatment during sensitization (OVA+CNP) had higher eosinophil and lymphocyte counts than their Air-exposed counterparts (p = 0.014). The number of lymphocytes was significantly increased in the NO_2_+OVA group, unlike the Air+OVA animals, where the lymphocyte counts did not increase significantly. CNP exposure by itself increased the number of neutrophils, without a significant difference between Air and NO_2_-exposed animals. The latter cells were significantly less abundant in the BAL fluids of the sensitized and CNP-treated animals (OVA+CNP). The percentage of alveolar macrophages was significantly increased in OVA-sensitized animals compared to Saline-treated controls, including in those receiving CNP treatment.

**Table 1:** BALF Cytology.

4-Week Air Exposure						
	Total cells	AM	Neutrophils		Lymphocytes	Eosinophils
**Saline**	2.18±0.68	2.03±0.69	0.03±0.01		0.04±0.01	0.08±0.03
	%	89.4±3.5	1.8±0.6		3.3±1.2	5.5±2.6
**CNP**	2.25±0.13	1.43±0.14	0.73±0.08^a^		0.08±0.01	0.01±0.00
	%	63.4±4.1^a^	32.6±3.7^a^		3.4±0.3	0.6±0.2
**OVA**	2.60±0.67	1.21±0.28	0.16±0.08^b^		0.08±0.02	1.16±0.37a,b
	%	47.0±1.7a,b	6.4±2.2^b^		3.0±0.5	43.5±2.9a,b
**OVA+CNP**	2.18±0.30	1.17±0.21	0.15±0.05^b^		0.22±0.04a,b,c	0.84±0.11
	%	48.3±4.1a,b	6.1±1.7 b		9.4±2.1a,b,c	36.2±4.2a,b,c
**4-Week NO_2_ Exposure**						
	**Total cells**	**AM**	**Neutrophils**	**Lymphocytes**		**Eosinophils**
**Saline**	0.75±0.11	0.63±0.1	0.05±0.01	0.03±0.01		0.05±0.01
	%	82.9±2.0	6.1±0.6	4.4±0.8		6.6±1.6
**CNP**	2.82±1.57	2.08±1.16^a^	0.63±0.36^a^	0.08±0.03		0.03±0.01
	%	72.7±1.9	22.5±1.9a,d	3.3±0.4		1.5±0.5
**OVA**	3.94±0.56	1.79±0.23	0.11±0.04^b^	0.17±0.04a,b,d		1.86±0.41a,b
	%	46.8±4.1a,b	3.2±1.0^b^	4.8±1.1		45.3±5.0a,b
**OVA+CNP**	3.47±0.71	1.38±0.33	0.08±0.02^b^	0.14±0.01a,d		1.87±0.46a,b,d
	%	40.2±4.3a,b	3.5±1.5 b	4.8±0.9^d^		51.6±5,4a,b,d

Values are means ± SE (n = 6 per group);

a p<0.05 vs. Saline within condition;

b p<0.05 vs. CNP within condition;

c p<0.05 vs. OVA within condition;

d p<0.05 vs. Air within treatment, by two way ANOVA; **AM**: alveolar macrophages. Exposure to NO_2_ alone did not change the profile of BAL cytology. OVA-sensitization significantly increased BAL eosinophil counts. CNP exposure by itself increased the number of neutrophils, without a significant difference between Air and NO_2_-exposed animals. Exposure to NO_2_ in OVA-sensitized animals significantly increased lymphocyte counts and tended to elevate BALF eosinophilia.

### Protein Expression of TH1, TH2 and TH17 Inflammatory Cytokines and IgE Levels

We investigated the effects of NO_2_ and CNP on the expression of TH1 and TH2 cytokines in both serum and BALF 24 h after the last challenge. In serum, the TH2 cytokines IL-4, IL-5 and IL-13, were significantly increased in the NO_2_+OVA group, but not in the NO_2_+OVA+CNP group, in which the level of theses cytokines were significantly lower than the NO_2_+OVA animals (Table 2).

**Table 2:** Serum Protein Levels of TH1 and TH2 Cytokines.

		TH1 Cytokines (pg/ml)				TH2 Cytokines (pg/ml)		
**4-Week Air Exposure**								
	**Total Protein (mg/ml)**	**IFN-γ**	**IL-1β**	**TNF-α**	**KC/GRO**	**IL-4**	**IL-5**	**IL-13**
**Saline**	38.1±2.3	19.8±3.2	85.7±17.3	53.0±16.8	336.6±131.9	1.2±0.3	50.1±7.5	0.0±0.0$
**CNP**	43.7±4.8	24.6±4.9	97.5±27.0	35.0±4.4	190.3±41.8	1.3±0.3	75.9±20.6	0.0±0.0$
**OVA**	27.2±1.6	24.9±12.4	147.7±64.2	58.6±12.4	731.1±309.4	1.7±1.2	81.2±46.2	1.2±1.2
**OVA+CNP**	28.5±3.8	9.2±4.3	66.8±13.0	63.8±16.8	603.4±82.1	0.5±0.1	53.7±9.1	0.1±0.1
**4-Week NO_2_ Exposure**								
	**Total Protein (mg/ml)**	**IFN-γ**	**IL-1β**	**TNF-α**	**KC/GRO**	**IL-4**	**IL-5**	**IL-13**
**Saline**	33.2±8.7	22.9±6.0	160.7±15.9	493.2±84.4^d^	1832.9±585.4	1.4±0.4	118.5±30.2	0.0±0.0$
**CNP**	48.3±5.4	159.3±63.7^ad^	142.2±25.2^c^	206.5±89.8^a^	1361.0±339.8	1.8±0.3	116.3±17.6	0.0±0.0$
**OVA**	39.3±4.1	44.1±9.4^b^	292.1±47.1^ad^	889.6±123.2^abd^	8329.9±1589.7^abd^	4.5±1.3^abd^	215.7±28.7^abd^	4.5±1.3^abd^
**OVA+CNP**	44.3±3.7^d^	32.8±5.2^b^	214.6±23.3^d^	822.0±127.1^abd^	5152.5±950.2^abcd^	3.0±0.6^d^	175.2±10.5^d^	1.0±1.0^c^

Values are means ± SE (n = 6);

a p<0.05 vs. Saline within condition;

bp<0.05 vs. CNP within condition;

c p<0.05 vs. OVA within condition;

d p<0.05 vs. Air within treatment, by ANOVA;

$ Below detection limit. In serum, the TH2 cytokines IL-4, IL-5 and IL-13, were significantly increased in the NO2+OVA group, but not in the NO2+OVA+CNP group. Exposure to NO_2_ significantly increased serum TNF-α expression, in all of the exposed groups, which was remarkably higher in both of the OVA-sensitized groups; NO2+OVA and NO2+OVA+CNP vs. NO2+Saline and NO2+CNP. In the NO2+OVA and NO2+OVA+CNP groups, the levels of IL1-b were significantly increased compared to their air-exposed counterparts. The combination of NO_2_ exposure and CNP treatment significantly increased the IFN-g levels; however, the level of this cytokine was again significantly lower in the NO 2+OVA+CNP group. The KC/GRO levels were increased following NO_2_ exposure, with substantial elevations in NO2+OVA and NO2+OVA+CNP groups compared to Air+Saline, with notable within-group variability.

As concerns the TH1 cytokines, exposure to NO_2_ significantly increased serum TNF-α expression, in all of the exposed groups. This expression was remarkably higher in both of the OVA-sensitized groups; NO_2_+OVA and NO_2_+OVA+CNP vs. the NO_2_+Saline (p = 0.005 and 0.008 respectively) and the NO_2_+CNP groups (p<0.001 and <0.001 respectively). In the former NO_2_+OVA and NO_2_+OVA+CNP groups, the levels of IL1-β were also significantly increased compared to their air-exposed counterparts (p = 0.004 and 0.002 respectively). The combination of NO_2_ exposure and CNP treatment significantly increased the IFN-γ levels; however, the level of this cytokine was again significantly lower in the NO_2_+OVA+CNP group. Finally, KC/GRO levels were increased following NO_2_ exposure, with substantial elevations compared to the Air+Saline group, in the NO_2_+OVA (p<0.001) and NO_2_+OVA+CNP (p<0.001) groups, with notable within-group variability.

In BALF, all groups taken together, in rats exposed to NO_2_, both KC/GRO (p = 0.005) and IL-5 (p = 0.009) cytokines were significantly increased compared air-exposed animals (Table 3). Individual increases in KC/GRO reached statistical significance vs. Air-Saline, only in the Air+CNP (p = 0.022), NO_2_+Saline (p = 0.018) and NO_2_+OVA (p = 0.013) groups.

**Table 3:** BALF Protein Levels of TH1 and TH2 Cytokines.

		TH1 Cytokines (pg/ml)				TH2 Cytokines (pg/ml)		
**4-Week Air Exposure**								
	**Total Protein (mg/ml)**	**IFN-γ**	**IL-1β**	**TNF-α**	**KC/GRO**	**IL-4**	**IL-5**	**IL-13**
**Saline**	1.7±0.5	1.9±1.0	92.1±10.8	37.9±14.6	1225.9±531.7	2.0±0.6	70.1±13.9	0.2±0.2
**CNP**	2.3±0.3	1.9±0.9	164.3±26.4	196.5±16.5	4612.5±801.5^ac^	2.2±0.5	98.5±18.9	0.1±0.1
**OVA**	3.1±0.3	1.4±0.9	171.4±56.8	217.4±80.2	1654.8±727.2	1.7±1.0	78.9±16.3	1.0±1.0
**OVA+CNP**	3.7±0.3^a^	2.5±1.2	219.3±58.9	144.5±63.2	2454.6±936.4	1.9±0.5	93.0±31.2	0.6±0.6
**4-Week NO_2_ Exposure**								
	**Total Protein (mg/ml)**	**IFN-γ**	**IL-1β**	**TNF-α**	**KC/GRO**	**IL-4**	**IL-5**	**IL-13**
**Saline**	3.1±1.1	2.6±1.1	144.9±20.2	162.6±40.7	3998.5±768.2^d^	2.7±0.6	148.1±30.1^e^	0.6±0.3
**CNP**	2.2±0.4	4.2±1.3	199.8±36.2	190.8±34.8	4814.9±764.7	3.4±1.0	127.8±24.4^e^	0.5±0.5
**OVA**	3.5±0.2	2.4±0.9	180.6±21.6	185.5±26.0	4576.8±830.8^d^	2.6±0.5	141.4±25.2^e^	0.3±0.2
**OVA+CNP**	4.1±0.4	2.6±1.1	200.6±47.1	173.1±63.4	3258.4±901.3	1.6±0.8	159.0±49.1^e^	2.6±1.8

Values are means ± SE (n = 6 per group);

a p<0.05 vs. Saline within condition;

b p<0.05 vs. CNP within condition;

c p<0.05 vs. OVA within condition;

d p<0.05 vs. Air within treatment, by ANOVA. NO_2_ exposure significantly increased both KC/GRO and IL-5 compared to air-exposed animals. Individual increases in KC/GRO were significantly higher vs. Air-Saline, in the Air-CNP, NO2+Saline and NO2+OVA groups.

**Figure 5:**
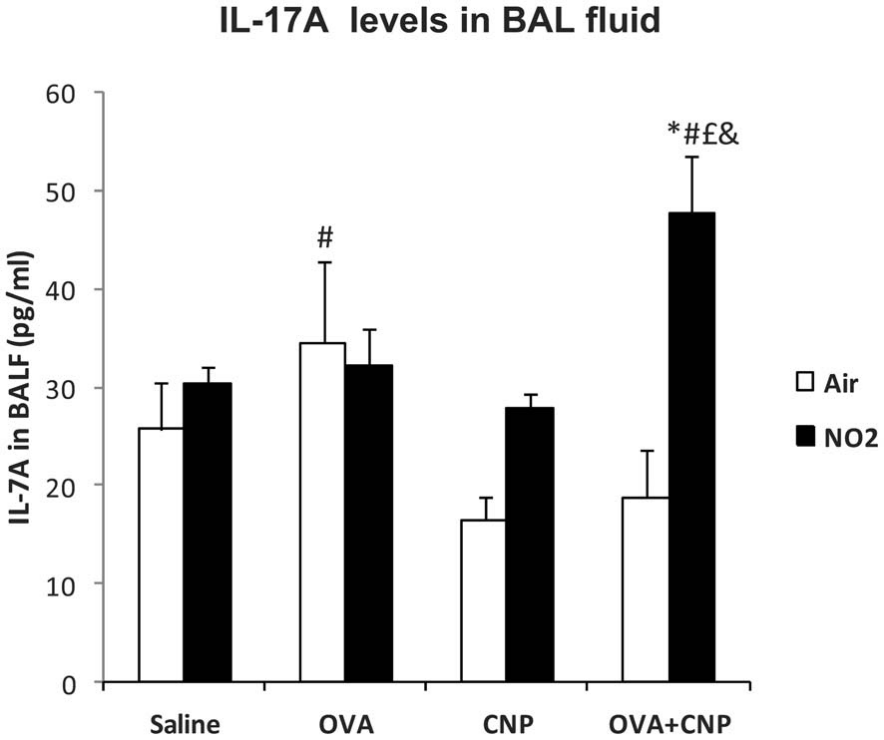
IL-17A levels in BAL fluid. Data are means ± SE; *: p<0.05 vs. Saline control within condition; #: p<0.05 vs. CNP within condition; £: vs. OVA within condition, &: vs. OVA within treatment, by ANOVA.

**Figure 6:**
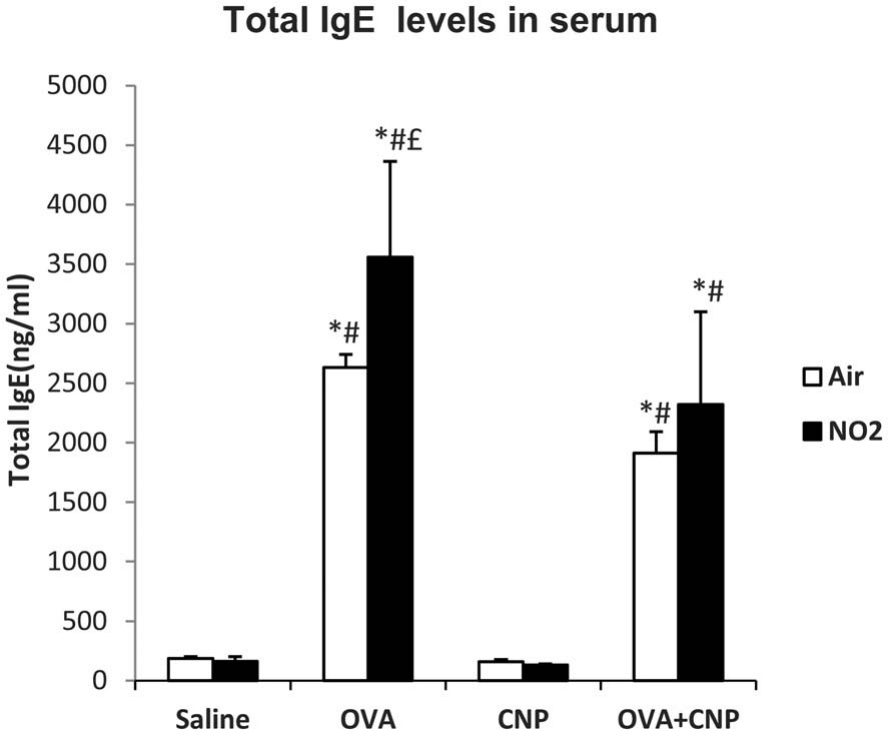
Total serum IgE levels. Data are means ± SE; *: p<0.05 vs. Saline control within condition; #: p<0.05 vs. CNP within condition; £: vs. OVA+CNP within condition, by ANOVA.

The levels of IL-17A in BALF are summarized in Figure 5. This cytokine was significantly increased in the NO_2_+OVA+CNP group, compared to Air+OVA+CNP (p<0.001), NO_2_+CNP (p = 0.008), NO_2_+OVA (p = 0.011) and NO_2_+Saline (p = 0.019) groups. Total serum IgE levels were significantly increased in all OVA-sensitized groups (Figure 6). And were significantly higher in the NO_2_+OVA compared to the NO_2_+OVA+CNP group (p = 0.025).

### Lung Histology

**Figure 7:**
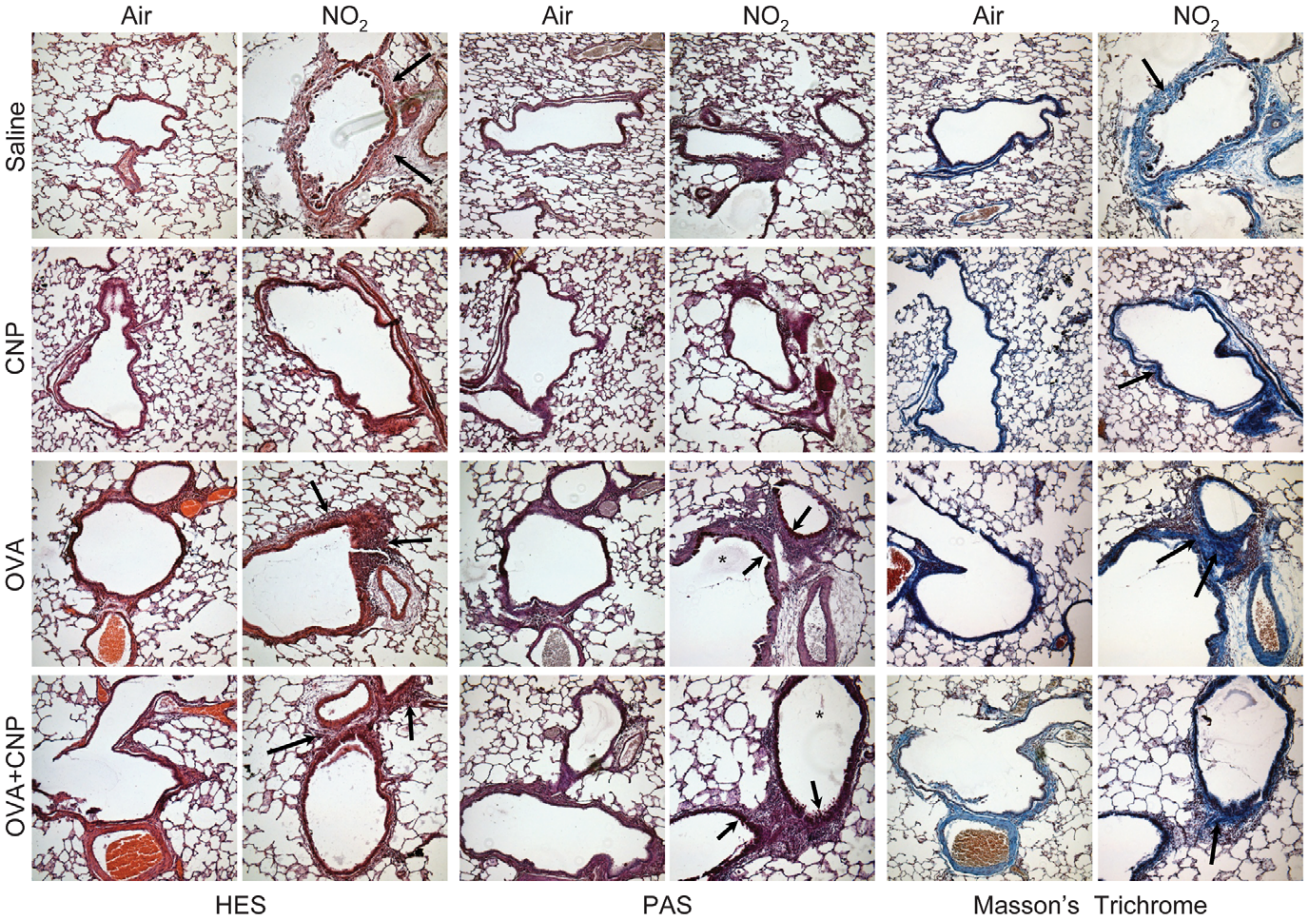
Representative histological images of HES (left columns), PAS (middle columns) and Masson’s trichrome stain (right columns). Original magnification: ×100. Exposure to NO_2_, resulted in inflammatory changes, including infiltration in the bronchial submucosa, perivascular areas and the surrounding alveolar septa (left columns: arrow). Mild to moderate goblet cell hyperplasia (middle columns: arrow), and the presence of mucus secretions inside the bronchial lumen (middle columns: star), following NO_2_ exposure in the OVA-sensitized groups. Increased collagen deposition in the bronchial wall associated with exposure to NO_2_ was found on Masson’s trichrome (middle columns: arrow).

In saline-treated air-exposed control (Air+Saline) animals, the bronchi, alveoli and airway epithelia were structurally intact without any signs of inflammation (Figure 7). Exposure to NO_2_, both in the Air+Saline and in the OVA-sensitized and challenged animals resulted in inflammatory changes, including inflammatory infiltration in the bronchial submucosa, perivascular areas and the surrounding alveolar septa. The cellular infiltrates mainly consisted of mononuclear cells and eosinophils. The inflammatory changes associated with NO_2_ exposure appeared more prominent in the OVA-sensitized groups. Furthermore, we found mild to moderate goblet cell hyperplasia on PAS stains, and the presence of mucus secretions inside the bronchial lumen, following NO_2_ exposure in the OVA-sensitized groups (Figure 7; middle columns). Masson’s trichrome staining (Figure 7; right columns) showed an increased collagen deposition in bronchial wall associated with exposure to NO_2_ in all groups. Increased collagen deposition and remodeling were more prominent in the OVA-sensitized animals. In the CNP-treated animals, histological examination showed the presence of clustered particles in the alveolar lumen, along with minimal histological changes.

## Discussion

The goal of this study was to assess the interaction of NO_2_, a gaseous air pollutant, and an elemental carbon nanoparticle, on inflammation, respiratory system mechanics and airway reactivity in a rat model of allergic airway inflammation. Although NO_2_ has previously been shown to enhance allergic lung inflammation and response to airway provocation [30], [31], the interactions of this known irritant with a carbon nanoparticle has not been previously assessed. The main findings of the present study were that: 1) 4 weeks of exposure to NO_2_ significantly enhanced allergic lung inflammation with increased BAL cell counts and both TH1 and TH2 serum cytokine expressions and airway reactivity to specific and non specific challenges in OVA-sensitized animals; 2) Exposure to CNP once per week during the NO_2_ exposure was characterized by increased neutrophils and alveolar macrophages in BALF along with increased TNF-α and IFN-γ expression in serum; 3) concomitant exposure to CNP during OVA-sensitization attenuated the airway reactivity to both methacholine and antigen, and lowered serum TH2 cytokines compared to NO_2_ exposure in OVA-sensitized animals alone; 4) allergen sensitization, but also exposure to CNP or NO_2_ reduced the rate of increase in EELV from 7 to 11 weeks, compared to saline-treated air-exposed controls, suggesting slowed lung growth.

In this study, we found that 6 hour daily, 5 days per week NO_2_ exposure at 10 ppm for 4 weeks during OVA sensitization and challenges significantly enhanced bronchial reactivity both to methacholine and antigen provocation. In the United States, the national Ambient Air Quality Standard for NO_2_ is 0.053 ppm (annual arithmetic mean concentration) [32]. Mean ambient indoor NO_2_ concentrations of approximately 0.032 ppm have been reported in Europe [33] and up to 0.54 ppm in developing countries [34]. Although the animals in this study were exposed to NO_2_ concentrations that were higher than the mean ambient indoor values reported in the literature, peak NO_2_ concentrations can be substantially higher in the vicinity of an indoor source, and can exceed 4 ppm [35]. Moreover, these higher concentrations are balanced by limited exposure duration in this study, which may underestimate long-term or life-long exposures to NO_2_ [8], [36]. We were not able to demonstrate increased responsiveness to methacholine in the OVA-sensitized and air-exposed group vs. the air-exposed saline group. The BN rat strain shows several features of allergic asthma in humans such as increased serum IgE levels, BALF eosinophils, and airway constriction in response to antigen inhalation, which were all present in the OVA-sensitized group in this study. However, increased airway responsiveness to methacholine is not systematically demonstrated in the literature [37]. Actually, this rat strain shows less airway responsiveness to cholinergic challenge than other rat species [38]. Also, lower doses of methacholine were used in our study, compared to other studies where increased airway responsiveness is usually observed with OVA-sensitization [39]. Finally, the repeated allergen challenges may have decreased airway hyperresponsiveness despite increased serum IgE and eosinophil counts in the BALF, as previously described in this model [40]. The finding that NO_2_ exposure increases bronchial responsiveness to methacholine is in agreement with previous studies in the literature. Poynter et al. found an increased airway reactivity to methacholine following a 6 hour daily, 3-day exposure to 25 ppm NO_2_, starting after immunization and challenge to OVA in mouse [31]. In the present study, NO_2_ exposure increased the serum levels of TNF-α, suggesting tissue macrophage activation. However, we did not find a concomitant increase in TNF-α levels in BALF. These results are different than in the study by Garn et al. where 20 days of continuous exposure to 10 ppm NO_2_ significantly decreased TNF-α protein levels in BALF and the ability of stimulated alveolar macrophages to release TNF-α, in Fischer rat [41]. No systemic measurements of TNF-α levels were reported in that study. Unlike the present study, in the study by Garn et al. the rats were exposed continuously to NO_2_. One hypothesis to explain the differences between systemic and BALF levels of TNF-α, is that the activation state may be different in alveolar macrophages compared to macrophages in other tissues. Macrophages can produce both pro- and anti-inflammatory mediators depending on the type of activation; a classical activation promotes the release of pro-inflammatory cytokines such as IL1-β and TNF-α, whereas an alternative activation leads to the release of inhibitory cytokines [41], [42]. Alveolar macrophages have been shown to be less potent in the production of inflammatory mediators [43]. Furthermore, chronic exposure to NO_2_ may act predominantly on alveolar macrophages in the lung to downregulate the production of pro-inflammatory cytokines [41].

The growth-related oncogene (GRO)-chemokine and its murine counterpart KC, belongs to a subset of CXC chemokines secreted by somatic cells, which includes human IL-8 [44]. The expression of this chemokine is induced in response to tissue injury and numerous inflammatory stimuli [45]. In vivo transgenic expression of this chemokine has been shown to mediate the attraction of neutrophils [46]. In this study, NO_2_ exposure increased the expression of this chemokine both in serum and BAL, the highest increases of which were observed in the OVA-sensitized group. However, a concomitant neutrophilia was not observed in the BALF in this particular group. The lack of neutrophilia in BALF after 4 weeks of exposure to NO_2_ may have been due to the kinetics of neutrophilic inflammation. It has been previously observed that although NO_2_ inhalation leads to neutrophilia for up to 3 days, the number of BALF neutrophils is significantly reduced after longer periods of exposure [41], [47]. This decrease may be due to the emergence of regulatory mechanisms such as inhibitory cytochine expression by alternative macrophage activation [48].

Nitrogen dioxide exposure increased lymphocyte counts in the BALF of OVA-sensitized animals in this study; however, it did not significantly increase the OVA-induced hypereosinophilia. In a previous study, Proust et al. found that, an acute 3-hour exposure to 20 ppm NO_2_ in BALB/c mice following allergen challenge, also induced significant airway hyperresponsiveness to methacholine, but was not accompanied by an increase in BALF hypereosinophilia produced by OVA-sensitization [49]. In another study, exposure to 2 ppm NO_2_ 24 hours before antigen challenge induced airway neutrophilia but did not alter airway reactivity [36]. In this study, the level of TH2 cytokines IL-4, IL-5 and IL-13 were significantly increased in OVA-sensitized animals exposed to NO_2_. These cytokines are known to contribute to allergic inflammatory processes, goblet cell hyperplasia, airway wall remodeling, and airway hyperresponsiveness [16]. These features were present on histological examination of lung samples of OVA-sensitized animals exposed to NO_2_ in this study (Figure 7). The discrepancy among the various studies in the literature is likely to be due to differences in concentration and duration of NO_2_ exposure, the timing relative to antigen immunization and challenge and the studied animal species and strain.

The levels of TH2 cytokines were not significantly increased in the Air+OVA group. The reason for the lack of more prominent elevations of TH2 cytokines in the Air-OVA group is not obvious but may have been due to the repetition of inhaled challenges that may have induced tolerance and attenuated the cytokine response [30]. Taken together, our data suggest that exposure to NO_2_ in OVA-sensitized animals, increased bronchial reactivity both to allergen and to non-specific airway challenge by methacholine, through a combined TH1 and TH2-type inflammatory cytokine expression. The fact that both serum TH2 cytokines and airway responsiveness were augmented in the NO_2_-exposed OVA-sensitized animals suggests a synergistic effect between sensitization and NO_2_ exposure. Although the intimate mechanism of this particular cytokine expression profile in response to NO_2_ exposure during antigen sensitization is not known, one hypothesis is that NO_2_-induced damage to the airway epithelium and increased permeability promotes the translocation of inhaled particulate antigen, thus increasing its bioavailability [50]–[52].

Ambient air can contain a complex mixture of gaseous and particulate pollutants produced by combustion. The interactions of these components both in modulating the allergic inflammatory response to allergen, and ultimately on airway responsiveness have rarely been assessed. Carbonaceous particles such as CNP represent one of the components of ambient particulates, with the advantage that they do not include adsorbed chemical substances, therefore allowing the study of the physical core of the particle itself [14]. We found that repeated CNP treatment in non-sensitized rats induced elevated neutrophil counts and increased the expression of KC/GRO in BALF. However, minimal changes in lung histology, and no difference in airway reactivity were observed. The lack of effect of CNP on airway reactivity in normal animals is in agreement with previous data in mouse [53]. A 7 hour inhalation of ultrafine (114 nm) carbon black particles has been shown to induce neutrophil recruitment in BALF 16 hours post-exposure, in wistar rat [54]. Li et al. also observed a neutrophilic recruitment in BALF with tracheal instillation of 14 nm but not with 260 nm carbon black particles at a similar dose than in the present study [22]. The combined exposure to NO_2_ and CNP increased the neutrophil and macrophage counts in BAL, and increased IFN-γ expression in serum. We are not aware of similar studies in the literature assessing the effect of combined NO_2_ and CNP.

In this study, the combination of repeated tracheal instillation of CNP during OVA sensitization and challenges did not significantly increase BALF hypereosinophilia, cytokine responses in BALF or serum, nor did it increase the airway responses to methacholine or antigen provocation, despite a slight but significant increase in lung tissue elastance in comparison to air-exposed OVA-sensitized animals. On the other hand, OVA-sensitized and challenged animals exposed concomitantly to NO_2_ and CNP showed reduced TH2 cytokine (IL-4, IL-5, IL-13) serum levels and airway responses to both non-specific and allergen provocation. This was a surprising result since other studies in mouse have shown an adjuvant effect of CNP on specific IgE production, when particles and antigen are co-administered [15], [55], [56]. Alessandrini et al. found that CNP inhalation prior to allergen challenge in Balb/c mice increases BAL cell infiltrate, TH2 cytokine production as well as airway responsiveness [53]. Conversely, Dong et al. found that although diesel exhaust particles (DEP) had adjuvant IgE activity in BN rats, DEP inhalation for 4 h per day on 5 consecutive days prior to allergen sensitization significantly attenuated the allergen-induced lung inflammatory responses [37]. However, in another study by the same group, 4 hours per day for 2 days of whole-body exposure to DEP 24 hours prior to allergen challenge showed increased airway reactivity to methacholine in BN rats [37]. On the other hand, DEP and carbon black particles may have distinct immunomodulatory effects in allergy. Van Zijverden et al. found that in Balb/c mice, DEP elicited a predominantly TH2 type response, whereas carbon black caused a combined TH1/TH2 response with increases in both IL-4 and IFN-γ [57]. In the present study, exposure to NO_2_ in CNP-treated animals produced a TH1-oriented cytokine response with elevations in IFN-γ and TNF-α. The reduction of TH2 cytokines in the same NO_2_ exposure in both CNP treated and OVA-sensitized animals may involve an immunomodulatory effect of CNP on allergic inflammation, in the BN rat model. The differences in the results of the present study and previous studies in the literature regarding the adjuvant role of CNP in allergic inflammation may be due to the chemical composition of the particle, but also dose, route of exposure (i.e.: inhaled vs. instilled) and timing of administration with respect to sensitization. Ultrafine CNP particles aggregate in solution and this may have significantly reduced their inflammatory potency [58]. Furthermore, repeated instillation of CNP may have induced adaptive mechanisms, thereby reducing the response to further inflammatory stimuli [59]. One possibility is that the tolerance seen in the NO2+OVA+CNP group compared to NO2+OVA is due to increases in regulatory T cells (Tregs) in the lungs. It has been shown that naïve T cells in the periphery can acquire immunosuppressive properties and become induced Tregs, which can inhibit hypersensitivity through the production of inhibitory cytokines and cellular interactions [60]. Yamashita et al. have shown that intravenous exposure to 165 nm crystalline C_60_ Carbon nanoparticles both inhibits delayed-type hypersensitivity and increases the ratio of Treg to total T (CD4+) cells in methyl-bovine serum albumin-sensitized mouse [61]. Furthermore, the significant elevation observed in IL-17A levels in BALF of the NO2+OVA+CNP group (Figure 5), can be involved in the modulation of the TH2 response to allergic sensitization [62]. Schnyder-Candrian et al. have previously demonstrated a dual role of this cytokine: although essential during antigen sensitization to establish allergic bronchial hyper responsiveness, IL-17 also attenuates the local production of the TH2 cytokines IL-4, IL-13, and IL-5 in the lung of OVA-sensitized mice [63]. Also, exogenous IL-17 reduced pulmonary eosinophil recruitment and bronchial hyperreactivity [63]. Moreover, the role of Tregs in the tolerance seen in the NO2+OVA+CNP group compared to NO2+OVA is further supported by the finding that under proinflammatory conditions, Tregs can promote the development of TH17 cells and production of IL-17 [64].

Comparison of EELV at 7 weeks of age, prior to any treatment or exposure, showed a significant increase at 11 weeks. In rat, beyond the initial period of rapid alveolarization that ends within 3 weeks of birth, the lungs show significant overall growth. Several studies have suggested that in this species, the number of alveoli still increases after the lung parenchyma reaches maturity [65]. This observation is in line with the data of Bolle et al. who found a 2-fold increase in EELV from age 5 to 13 weeks in Wistar Kyoto rats, although this rate of growth may be different in other rat strains [66]. In the present study, the rate of increase in EELV was significantly slowed down by NO_2_ exposure, but also by sensitization to allergen, CNP treatment or both (Figure 5). There were no significant differences in body weight between the air and NO_2_-exposed groups, suggesting that both allergic inflammation and exposure to pro-inflammatory gaseous (NO_2_) or particulate (CNP) pollutants may significantly reduce the rate of overall lung growth beyond the “bulk alveolarization” stage in BN rat. Alternatively, reduced EELV could be due to alterations in gas exchange increasing ventilatory demand. Nevertheless, this outcome is interesting in light of existing data demonstrating that both pre and post-natal exposure to air pollution decrease lung growth in children. [67], [68].

In conclusion, in this study we assessed the effect of NO_2_ exposure and its interaction with CNP in allergen-sensitized BN rat, a model of allergic asthma. Our findings demonstrate that exposure to NO_2_, a gaseous air pollutant, significantly enhances lung inflammation and airway reactivity, with a significantly larger effect in animals sensitized to allergen and challenged during exposure to NO_2_, which was associated a higher expression of both TH1 and TH2-type cytokines. Conversely, exposure to NO_2_ in animals undergoing repeated tracheal instillation of CNP alone, increased BALF neutrophilia and enhanced the expression of TH1 cytokines: TNF-α and IFN-γ, but did not show an additive effect on airway reactivity in comparison to NO_2_ alone. The exposure to NO_2_ combined with CNP treatment and allergen sensitization however, resulted in a significant decrease in both airway reactivity to allergen and to methacholine, and a reduction in TH2-type cytokines compared to allergen sensitization alone, suggesting an immunomodulatory effect of repeated tracheal instillation of CNP on the proinflammatory effects of NO_2_ exposure in sensitized BN rat. Furthermore, our findings suggest that NO_2_, CNP and OVA sensitization may significantly slow lung growth in proportion to body size, in parenchymally mature animals, and may provide a model for the study of the adverse effects of environmental pollutants on lung development in asthma.
