# Correction: Effet of Combined Nitrogen Dioxide and Carbon Nanoparticle Exposure on Lung Function During Ovalbumin Sensitization in Brown Norway Rat

**DOI:** 10.1371/annotation/d271d9c1-5588-4b43-85c3-d3de58ab61a4

**Published:** 2013-05-17

**Authors:** Skander Layachi, Françoise Rogerieux, Franck Robidel, Ghislaine Lacroix, Sam Bayat

There were multiple errors in the article.

The title of the article was incorrect.

The correct version is: Effect of Combined Nitrogen Dioxide and Carbon Nanoparticle Exposure on Lung Function During Ovalbumin Sensitization in Brown Norway Rat

The correct citation is: Layachi S, Rogerieux F, Robidel F, Lacroix G, Bayat S (2012) Effect of Combined Nitrogen Dioxide and Carbon Nanoparticle Exposure on Lung Function During Ovalbumin Sensitization in Brown Norway Rat. PLoS ONE 7(9): e45687. doi:10.1371/journal.pone.0045687

The Table 1 legend was incorrect. The correct version is:

**Table 1.** Values are means (×106 cells) ± SE (n = 6 per group); a: p<0.05 vs. Saline within condition; b: p<0.05 vs. CNP within condition; c: p<0.05 vs. OVA within condition; d: p<0.05 vs. Air within treatment, by two way ANOVA; AM: alveolar macrophages. Exposure to NO2 alone did not change the profile of BAL cytology. OVA-sensitization significantly increased BAL eosinophil counts. CNP exposure by itself increased the number of neutrophils, without a significant difference between Air and NO2-exposed animals. Exposure to NO2 in OVA-sensitized animals significantly increased lymphocyte counts and tended to elevate BALF eosinophilia.

Figure 5 was incorrect. The correct version can be found here: 

**Figure:**
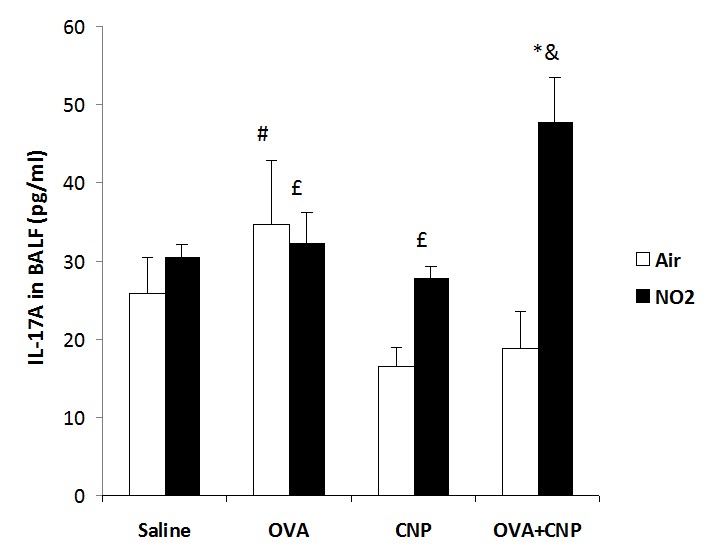


The Figure 5 legend was incorrect. The correct version can be found here:

**Figure 5. IL-17A levels in BAL fluid.** Data are means ± SE; *: p<0.05 vs. Saline control within condition; #: p<0.05 vs. CNP within condition; £: vs. OVA within condition, &: vs. Air within treatment, by ANOVA. 

